# On the Treatment of Dysentery

**Published:** 1852-07

**Authors:** Robert Durrett

**Affiliations:** Resident Graduate at the Louisville Marine Hospital; Louisville


Art. II.—*On the Treatment of Dysentery.* By Robert Durrett, M. D., Resident Graduate at the Louisville Marine Hospital, during the year 1851.

There were admitted into the Louisville Marine Hospital, in 1851, forty-four cases of dysentery, of which number eighteen cases of an acute character were submitted to the saline treatment, in all of which except one it proved successful. The causes of failure in this case were undoubtedly the advanced stage of the disease and the extreme feebleness of the patient. The mode of treatment was as follows. The patient, according to his physical strength and habits, was ordered sulphate of magnesia, from one to four drachms every hour, till serous evacuations were produced, free both from blood and mucus. The patient was then ordered a Dover's powder, and generally an enema of water and morphia. It was not found necessary to repeat the sulphate of magnesia after the first day in any but a very few cases.

After the administration of the opiates, the patient usually fell asleep, and awoke declaring himself relieved, and continued to improve under the administration of an occasional opiate. Enemas of spirits of turpentine ℥ss, mixed with an egg, repeated once or twice a day, or oftener if the severity of the symptoms demanded it, had the happiest effect in six cases. When there was much tenesmus, a drachm of tinct. opium was combined with the turpentine and egg. The patient frequently retained this injection without complaint for several hours, and almost invariably passed it off either with a good discharge from the bowels, or one was sure to follow immediately. In all cases of dysentery, where the inflammation does not extend beyond the rectum, I should be disposed to give the turpentine enema a trial. Many cases of chronic dysentery were treated satisfactorially with copper and opium. Some cures were very happily effected with this article, in which time-honored remedies failed.

Louisville, June, 1852.

